# Report of Three Peculiar Cases of Paralysis, with Recovery in Each Case

**Published:** 1881-03

**Authors:** H. D. Valin

**Affiliations:** 802 S. Halsted St., Chicago


### ARTICLE V.

### REPORT OF THREE PECULIAR CASES OF PARALYSIS, WITH RECOVERY IN EACH CASE. By H. D. Valin, M.D., Chicago. (Read before a meeting of the West Chicago Medical Society, January 24, 1881.)

CASE I. *Paralysis of Lower Extremities, with Tremors and Convulsions Following a Concussion of the Spinal Cord.*

W. H., male, single, aged twenty-four, born in this country, heavily built, strong and plethoric. Family healthy; no constitutional disease. While walking very fast, on a wooden sidewalk in the city, November 14, 4 p.m., came across the space left by a missing plank which his left leg entered, and, with an effort to prevent a fall forward, he fell flat on his back on the sidewalk, producing concussion of the spinal cord.

There was complete paralysis of the lower extremities, with anæsthesia, cold clammy skin, retention of urine, slow pulse, and respiration increased in frequency and labored. The left arm was slightly affected, and there was dizziness and headache. No external injury. Pain excessive in the lumbar region and reflected over the abdomen.

A physician, called on the spot, diagnosed concussion of the

---

spinal cord, and proposed an hypodermic injection of morphia, but the patient and his friends refused, and the patient was taken home in a street car. I saw the case at 7:30 p. m., and prescribed a liniment:

℞ Chloroform.................................℥ss.

Morphiæ sulphat..............................gr. ij.

Tr. opii.....................................

Tr. belladonnæ...............................*a a* ʒij.

Liniment. saponis............................ad ℥iij.

M.

To be applied all over the back every half hour. Patient took, also, three-eighths grain of morphia (powder) every hour to induce sleep. (Three powders were taken.) Directions were left to warm the feet and legs. Patient slept about half an hour that night. Pain mostly relieved. Micturition took place, though with difficulty, the next morning.

A good deal of pain was complained of in the head, in the pit of the stomach, over the solar plexus of nerves, and also in the lungs. Bowels had not moved for two days. He took 10 gr. each of calomel and ipecac which moved the bowels thoroughly the next day and produced emesis, once.

November 18, he began to take 30 grs. iodide of potassium and 50 grs. of the bromide in twenty-four hours, and took that during nine days. He began to improve a little from the beginning. Headache diminished, and so the pain in the back, while some motion returned in the right leg. He had bromism on fourth day, on which account he had to diminish the dose of his remedy. He used to take three-eighths grain morphia at night, sometimes twice as much. Sensibility returned partly in the legs, more in the right, also a slight amount of motion. Less pain was complained of, though there was a good deal of tenderness along the spine, and over the abdomen. Appetite increased. There was all that time some tympanitis of the bowels, for which he used synapisms, oil of turpentine stupes, and castor oil internally.

From the 22d to the 29th of November I was absent, and found the patient about the same on my return. He had not taken any remedy for three days; had been feverish, and had had chills two or three times. He used to have involuntary tremors of the legs in the night. I prescribed a few doses of 8 grs. quiniæ and 5 grs. comp. ipecac. powders, and bromide of potassium, 40 grs. a day, with 1½ drachms of fluid extr. of ergot. The morphia powders were replaced by the deodorized tr. of opium in equivalent doses, and this was taken now and then when required.

It was eighteen days after his fall when the patient was able to get up once a day with crutches. Sensibility was so blunt in the left leg that the prick of a pin would hardly be noticed.

December 16. Patient had not taken any remedy for a few days, and had moved with his family to the next block on the day previous, catching a bad cold. About 3 p. m., he fell into a sort of tonic spasm, with tremors of the lower extremities, fearful headache, contraction of the pupils, and stiffness of the neck and whole vertebral column. I saw him about 4:30 p. m., when he began to have clonic convulsions, and a wild delirium, tearing his bedclothes, making violent motions, and holding a pillow between his arms with efforts at tearing it with his teeth. The pulse was increased to over a hundred pulsations during the attacks, each of which lasted about twenty minutes, during which most of the muscles would strongly contract, and the limbs be partially flexed. The scalp and integument of the back were hot and tender, every passive movement being painful; but the control of the will had a certain influence over the convulsions at the beginning.

I applied ice to the head, gave about 40 drops of the deodor. tr. of opium, half a drachm bromide of potassium, and administered chloroform on a handkerchief at every return of the convulsions, that is six different times. As the patient had not passed water since morning, and as the bladder was painful under pressure, a warm compress was applied over it, and micturition took place fifteen minutes later with some difficulty. There was no albumen in the urine. It was half past six o'clock, and the patient being conscious, the chloroform was stopped—one ounce had been used—and there were no more convulsions that day. Patient took quinine for his cold, bromide of potassium, and 20 drops of the deod. tr. of opium, every three hours.

Patient improved till December 22, when he had convulsions more violent than the first time, and he remained in that state from 4 to 7 p. m. Again he inhaled chloroform ℥jss, and he took half a drachm each of chloral hydrate and bromide of potassium. The convulsions ceased after he was under the full influence of the chloral and fast asleep. In his delirium he sang mourning hymns, and his hearing was good, at least in regard to musical notes, for a hand-organ happening to play in the street, he made the motion of turning the handle with his right hand, and tuned his groans to the music. Between each convulsion he was rational. He again complained of a desire to micturate. During these convulsions, they referred to his having been bitten on the hand by a dog three years ago, and he alluded to it himself, but he had no difficulty in drinking water, and no sign of hydrophobia. These convulsions must have been the result of cold on the hyperæmic spinal cord and membranes, manifesting thus all the signs of cerebro-spinal meningitis. Patient's bed lay in a corner of the room, with a door at his feet, and one near his head, communicating with unheated rooms which were often opened into, sometimes both at the same time.

December 24. He began to take the sulphate of strychnia one-tenth gr. three times a day. This afforded him much relief, and he is able to be out now every day with his crutches. The spinal column is tender in the lumbar region, and the left leg is too weak to bear the body's weight; it is yet hardly sensitive to external irritation. Patient is nervous and irritable.

This case belongs to the third category of cases as given by Erb in Ziemssen's Cyclopædia, and an impairment of the lower extremities is likely to remain for life. My treatment was that of Erb just referred to, except that he says: "*Strychnia should not be resorted to until all the symptoms of irritation are past.*" But in this peculiar case, I am glad that I did not delay its use so long. I propose now to employ electricity. I may add that, a few days ago, patient took by mistake 50 grs. of bromide of potassium in the evening. The result was that he hardly slept any that night, was restless, vomited twice, had roaring in the ears, and a loss of appetite. These symptoms disappeared the next day.

CASE II. *Hemiplegia at fourth month of pregnancy.*

December 18, 1880. Mrs. L., at fourth month of pregnancy, fell unconscious and paralyzed, on getting ready to sweep the floor in the morning. Her mother died from paralysis. She is thirty-two years old, tall and spare, of the nervous temperament, and was married last year, this being the first time that she has become pregnant. Her husband left for Texas two weeks previous, and she had much grief on that account. Following the fit, patient's left side was completely paralyzed; the left side of the tongue, the left arm and the left leg not being at all influenced by any voluntary effort. Patient was at first unable to speak, had difficulty in swallowing, and did not sleep for seventy-two hours, or until morphine was resorted to. The sensibility was normal in the paralyzed limbs, and no pain was complained of anywhere.

I saw her on the third day after the accident. She could speak with difficulty, could swallow fluids, a little at a time, and could move the leg slightly. The tongue was protruded with difficulty, and to the left side; it was coated yellowish. Pulse was 92 a minute, temperature normal; the bowels had not moved for two days, and the uterus was normal as to its shape and position, at the present period of pregnancy. I ordered a tablespoonful of castor oil, repeated after three hours, which had no effect, and I gave 10 grs. bromide of potassium in a drachm of co. tr. of cinchona every four hours, during three days, to calm the nervous excitement and tone the system; I also gave one-fourth gr. morphia powders to procure sleep. December 21, I replaced the bromide by one-sixteenth gr. of strychnia three times a day. A fly blister two inches square was applied to the right side of the neck on some painful points, and to make counter-irritation. Patient had to take morphia to induce sleep for five nights, and she took, December 22, nine co. rhubarb pills, in divided doses to move her bowels. From that day rapid improvement set in. Motion returned in the leg, in the arm also, slightly at first, and the hand began to grasp a little. The tongue cleared in a couple days, and the appetite returned. Patient's husband came back about that time, and she was able to be up, when supported on both sides.

December 28, 4 grs. of citrate of iron were added to the strychnia three times a day. A fly blister three inches square was applied over the roots of the left brachial nerves. This seemed also to have had a good result. Motion became more and more perfect in the arm. January 16. Patient is perfectly well, and resumes her daily occupations. Disease did not interfere with the pregnancy.

CASE III. *Bulbar paralysis recurring after one year. No treatment.*

H, male, single, aged seventy-two years; was living in a mountainous district, alone in a stone house, where he was much exposed to cold. It was in the winter of 1878, about the 15th of December; on rising in the morning he noticed that he could not speak at all, though his memory of words was not impaired, and that he could hardly swallow. He belongs to a healthy family, never had any constitutional taint, but has atony of the bladder, with incontinence of urine at times. He always had some difficulty of speech, depending on a slight degree of tongue-tie.

On the third day he could say a few words, and swallow better. The left side of the tongue was paralyzed and that organ was protruded to the left side, motions of the head were slightly impaired, and patient was very emaciated. Pulse 82 a minute, no fever, some winter cough. He had suffered from want, cold and privations, anxieties and depression. He had some wealth, and had just lost several hundred dollars in transactions. A physician prescribed 5 grs. bromide of potassium three times a day, which did not seem to have any effect, and he was convalescent all winter and got better in March. He attended to a part of his affairs during that while.

November 1879, patient had another attack for which he took 10 grs. bromide of potassium three times a day for a week, and afterward, the citrate of iron and strychnia in appropriate doses. He improved in a few weeks, but did not feel perfectly well before spring. He has had no return of the disease yet this winter.

802 S. Halsted St., Chicago.

